# 2125. Activity of Novel β-Lactam/β-Lactamase Inhibitor Combinations against Serine-Carbapenemase producing Carbapenem-Resistant *Pseudomonas aeruginosa*

**DOI:** 10.1093/ofid/ofad500.1748

**Published:** 2023-11-27

**Authors:** Su Young Lee, Christian M Gill, David P Nicolau

**Affiliations:** West Coast University, Los Angeles, California; Hartford Hospital, Hartford, Connecticut; Hartford Hospital, Hartford, Connecticut

## Abstract

**Background:**

Antimicrobial resistance in *Pseudomonas aeruginosa* is complex and multifaceted. While the novel β-lactamase inhibitors (BLI), avibactam, relebactam, and vaborbactam inhibit serine-based β-lactamases, the comparative potency of the novel β-lactam (BL) /BLI combinations against serine-carbapenemase producing *P. aeruginosa* is unknown. The present study sought to compare the *in vitro* activity of ceftazidime/avibactam, ceftazidime, imipenem/relebactam, imipenem, meropenem/vaborbactam, and meropenem against serine-β-lactamase producing *P. aeruginosa*.

**Methods:**

Carbapenem-resistant *P. aeruginosa* were collated through the Enhancing Rational Antimicrobials against Carbapenem-resistant *P. aeruginosa* (ERACE-PA) Global Surveillance. Isolates positive for serine-based carbapenemases were assessed. Minimum inhibitory concentrations (MICs) were determined by broth microdilution to each novel-BL/BLI and BL alone. *In vitro* potency was assessed by MIC_50/90_ and percent of isolates susceptible per CLSI guidance.

**Results:**

GES was the most common carbapenemase identified (n=59) followed by KPC (n=8). Ceftazidime/avibactam had MIC_50_/MIC_90_ values of 4/8 mg/L and 91% of isolates were susceptible. Conversely, ceftazidime alone was active against only 3% of isolates. The MIC_50_/MIC_90_ of imipenem/relebactam were 16/ >16 mg/L and 13% of all isolates were defined as susceptibility. Against the KPC-producing isolates, 38% were susceptible to imipenem/relebactam compared with 0% for imipenem. The meropenem/vaborbactam MIC_50_/MIC_90_ were >16/ >16 mg/L, and 6% of isolates were susceptible which was similar to meropenem alone (MIC_50_/_90_, >8/ >8 mg/L; 3% susceptible) suggesting the addition of vaborbactam cannot overcome co-expressed, non-enzymatic resistance mechanisms.

**Conclusion:**

Among the novel BL/BLIs, ceftazidime/avibactam displayed better *in vitro* activity and thus is a rational treatment option for serine-carbapenemase harboring *P. aeruginosa*. While imipenem/relebactam displayed some activity particularly against isolates with *bla_KPC_*, meropenem/vaborbactam exhibited poor activity with MICs similar to meropenem alone. These data can be integrated with rapid molecular diagnostics to guide empiric therapy.

**Disclosures:**

**Christian M. Gill, PharmD**, Cepheid: Grant/Research Support|Entasis therapeutics: Grant/Research Support|Everest Medicines: Grant/Research Support|Shionogi: Grant/Research Support **David P. Nicolau, PharmD**, Allergan: Advisor/Consultant|Allergan: Grant/Research Support|Cepheid: Advisor/Consultant|Cepheid: Grant/Research Support|Merck: Advisor/Consultant|Merck: Grant/Research Support|Pfizer: Advisor/Consultant|Pfizer: Grant/Research Support|Shionogi: Advisor/Consultant|Shionogi: Grant/Research Support|Tetraphase: Advisor/Consultant|Tetraphase: Grant/Research Support|Venatorx: Advisor/Consultant|Venatorx: Grant/Research Support|Wockhardt: Advisor/Consultant|Wockhardt: Grant/Research Support

